# Elektrokonvulsionstherapie in der Schwangerschaft: Fallbericht und interdisziplinäre Behandlungsvorschläge

**DOI:** 10.1007/s00115-020-00960-7

**Published:** 2020-07-17

**Authors:** David Zilles-Wegner, Sarah Trost, Karoline Walliser, Leif Saager, Sebastian Horn, Mareike Ernst

**Affiliations:** 1 Klinik für Psychiatrie und Psychotherapie, Universitätsmedizin Göttingen, Von-Siebold-Str. 5, 37075 Göttingen, Deutschland; 2 Klinik für Anästhesiologie, Universitätsmedizin Göttingen, Göttingen, Deutschland; 3 Klinik für Kinderheilkunde und Jugendmedizin, Pädiatrische Kardiologie, Intensivmedizin und Pneumologie, Universitätsmedizin Göttingen, Göttingen, Deutschland; 4 Klinik für Gynäkologie und Geburtshilfe, Universitätsmedizin Göttingen, Göttingen, Deutschland

**Keywords:** Anästhesie, Geburtshilfe, Neonatologie, Wirksamkeit, Sicherheit, Anesthesia, Obstetrics, Neonatology, Effectiveness, Safety

## Abstract

**Hintergrund:**

Psychische Störungen in der Schwangerschaft sind häufig. Besonders bei schweren affektiven oder psychotischen Störungen mit Notwendigkeit eines raschen Ansprechens kann eine Elektrokonvulsionstherapie (EKT) indiziert sein. Dazu vorliegende Übersichtsartikel unterscheiden sich methodisch stark, was zu unterschiedlichen Schlussfolgerungen hinsichtlich der Anwendung der EKT bei Schwangerschaft führt.

**Ziel der Arbeit:**

Darstellung eines neuen klinischen Falls sowie interdisziplinärer Behandlungsvorschläge zur sicheren Anwendung der EKT bei Schwangerschaft.

**Methoden:**

Fallbericht und selektive Literaturübersicht unter besonderer Berücksichtigung der existierenden systematischen Reviews.

**Ergebnisse und Diskussion:**

Die aktuelle Kasuistik zeigt die potenziell hohe Wirksamkeit sowie die für Mutter und Fetus sichere Anwendung der EKT während der Schwangerschaft. Die in der Literatur beschriebenen unerwünschten Ereignisse entsprechen qualitativ weitgehend den Risiken bei schwerer psychischer Störung in der Schwangerschaft. Zur besseren Nutzen-Risiko-Abwägung wären größere Fall-Kontroll-Studien wünschenswert. Bei sorgfältiger Indikationsstellung, guter interdisziplinärer Abstimmung und Beachtung der Besonderheiten in der praktischen Durchführung ist die EKT auch in der Schwangerschaft eine sinnvolle Behandlungsoption.

## Abstract (en)

**Background:**

Psychiatric disorders during pregnancy are common. Electroconvulsive therapy (ECT) can be indicated in severely affective or psychotic disorders with the necessity of a rapid response. Currently available review articles greatly differ in the methodology, leading to divergent conclusions concerning the use of ECT during pregnancy.

**Objective:**

Description of a new clinical case and interdisciplinary treatment suggestions for the safe application of ECT in pregnancy.

**Methods:**

Clinical case report and selective review of the literature with special consideration of existing systematic reviews.

**Results and conclusion:**

This case report shows the potentially high effectiveness and safe administration of ECT in pregnancy for both mother and fetus. The undesired adverse events associated with ECT described in the literature are largely qualitatively congruent with the risks of severe psychotic disorders in pregnancy per se. For a better risk-benefit analysis, larger case control studies would be desirable. Under the premise of a thorough evaluation of the indications, good interdisciplinary coordination and consideration of the specific practical requirements, ECT is a useful therapeutic option in pregnancy.

## Hintergrund

Schwerwiegende psychische Erkrankungen in der Schwangerschaft stellen einen relevanten Risikofaktor für Geburtskomplikationen dar. Nach einer aktuellen Registerstudie sind schizophrene und affektive Psychosen während der Schwangerschaft mit einer Vielzahl von mütterlichen, fetalen und neonatalen Komplikationen verbunden [28]. Neben immanent psychiatrischen Aspekten wie Suizidalität mit ggf. erweitertem Suizid sowie einer gestörten Mutter-Kind-Bindung umfassen diese ein erhöhtes Risiko für Sectio, ante- und postpartale Blutungen, vorzeitige Plazentalösung, Frühgeburten, Totgeburten und fetale Auffälligkeiten wie Wachstumsrestriktion und chronischer fetaler Stress.

Die Behandlung der psychischen Störung muss bei vorliegender Schwangerschaft in besonderem Maße Verträglichkeit und Sicherheit für Mutter und ungeborenes Kind gewährleisten. Zur Pharmakotherapie sind dazu Datenbanken wie etwa die des Pharmakovigilanz- und Beratungszentrums für Embryonaltoxikologie (www.embryotox.de) verfügbar, die auf Grundlage von Fallberichten und -serien spezifische Empfehlungen zu einzelnen Wirkstoffen geben.

In manchen Fällen akuter psychiatrischer Störungen in der Schwangerschaft ist eine Psychopharmakotherapie nicht möglich, nicht gewünscht oder aber nicht ausreichend wirksam, sodass bei gegebener Indikation basierend auf entsprechenden Leitlinienempfehlungen [5–7] eine Elektrokonvulsionstherapie (EKT) in Betracht gezogen werden kann. Nationale wie internationale Leitlinien berücksichtigen die EKT in ihren Empfehlungen bei schwangeren Patientinnen und entsprechendem Schweregrad der Erkrankung. Die NICE-Guideline „Antenatal and postnatal mental health“ etwa empfiehlt die EKT bei schwerer Depression, Manie, Mischzuständen oder Katatonie und zugleich bestehendem gesundheitlichem Risiko für Mutter oder Fetus [19]. Diese Empfehlungen basieren auf Fallsammlungen, die mangels der Möglichkeit randomisierter, kontrollierter Studien bei Schwangeren die bestmögliche Evidenz darstellen.

Die Indikationsstellung zur EKT in der Schwangerschaft ist trotz der Leitlinienempfehlungen mit Unsicherheiten verbunden und erfolgt daher nur selten. 2008 wurden für Deutschland lediglich fünf Behandlungen bei Schwangeren beschrieben [16]. Dies mag auch damit zusammenhängen, dass bisherige Übersichtsarbeiten zu sehr unterschiedlichen Schlussfolgerungen hinsichtlich der Sicherheit der EKT bei Schwangeren gelangen [23]. Tab. 1 fasst die beschriebenen unerwünschten Ereignisse ohne Kausalitätsbeurteilung zusammen.

**Table 1:** 

Mutter	Fetus/Neugeborenes
Prolongierte Anfälle	Bradykardie/Arrhythmie
Uteruskontraktionen ohne Frühgeburt	Frühgeburt
Abdominelle Schmerzen	Abort/Totgeburt
Vaginale Blutung	Kongenitale Anomalien
Plazentalösung	Intrauterine Wachstumsrestriktion
Hämaturie	Neonatales Atemnotsyndrom
Präeklampsie	Mentale Retardierung
Sectio	

In einschlägigen Publikationen zur Therapie depressiver Störungen in der Schwangerschaft findet die EKT zum Teil keine Erwähnung [24]. In der deutschsprachigen Literatur fehlen konkrete Vorschläge zum praktischen Vorgehen [2], lediglich eine neuere internationale Publikation [26] beinhaltet Empfehlungen für das interdisziplinäre Management betroffener Patientinnen.

Ziel der aktuellen Arbeit ist es daher, basierend auf unserem konkreten Vorgehen im Einzelfall sowie den verfügbaren Literaturübersichten interdisziplinäre Vorschläge für die sichere Anwendung der EKT bei schwangeren Patientinnen zu erstellen.

## Methoden

Neben der Darstellung des klinischen Falls werden Behandlungsvorschläge zum Vorgehen bei EKT in der Schwangerschaft aus den Fachdisziplinen Psychiatrie, Geburtshilfe, Anästhesiologie und Neonatologie gegeben. Diese haben nicht den Stellenwert von Leitlinienempfehlungen, sondern wurden im interdisziplinären Austausch aus der verfügbaren internationalen Literatur sowie den eigenen Erfahrungen im Sinne der guten klinischen Praxis abgeleitet.

## Fallbericht

### Psychiatrie

Die 18-jährige schwangere Patientin wurde mit einem schizomanischen Syndrom aufgenommen. Psychopathologisch bestand eine Wahnsymptomatik mit hoher Wahndynamik einschließlich einer Negierung der Schwangerschaft, formalgedanklich Ideenflucht und Gedankenabreißen, zum Teil Personenverkennungen und Ich-Störung im Sinne von Gedankeneingebungen. Der Affekt war teilweise aggressiv-gereizt, das Verhalten distanzlos, zum Teil bizarr, psychomotorisch agitiert mit reduziertem Schlafbedürfnis sowie Konzentrations- und Gedächtnisstörungen.

Es wurde eine Medikation mit Quetiapin 700 mg/d sowie 30 mg/d Diazepam etabliert. Dennoch kam es zu aggressiven Erregungszuständen mit Gefährdung des ungeborenen Kindes, indem die Patientin sich auf den Bauch schlug, sich auf den Bauch warf oder diesen gegen Wände quetschte. Es erfolgte die Unterbringung nach Betreuungsrecht. Mehrfach waren Fixierungsmaßnahmen und intramuskuläre Medikation erforderlich.

Aufgrund der Notwendigkeit eines raschen Ansprechens sowie schwangerschaftsbedingt eingeschränkter Pharmakotherapie stellten wir die Indikation zur EKT. Patientin und gesetzlicher Betreuer willigten in die Behandlung ein. Ab der vollendeten 28. Schwangerschaftswoche (SSW) erfolgten neun EKT-Behandlungen (3/Woche, bitemporale Elektrodenplatzierung) in Anwesenheit von Gynäkologie/Geburtshilfe, Anästhesiologie und Neonatologie. Hierunter kam es zu einer raschen Besserung der Psychopathologie. Psychotische Symptome, psychomotorische Unruhe und aggressives Verhalten nahmen ab, Fixierungsmaßnahmen waren nicht mehr erforderlich. Ein einmalig prolongierter Anfall unter EKT wurde mittels Midazolam komplikationslos beendet. Parallel dazu kam es einmalig zu einer selbstlimitierenden fetalen Bradykardie (siehe unten). Die Patientin berichtete subjektiv über leichte Gedächtnisstörungen. Weitere unerwünschte Wirkungen traten nicht auf.

Der Versuch einer pharmakologischen Erhaltungstherapie schlug fehl, da unter Quetiapin 1000 mg/d keine ausreichende Konzentration im therapeutischen Drug Monitoring erreicht wurde, am ehesten aufgrund schwangerschaftsassoziierter CYP3A4-Induktion [20, 27]. Parallel zur Umstellung auf Risperidon 7 mg/d erfolgten vier weitere EKT-Behandlungen, die letzte mit 33 SSW. Das schizomanische Syndrom war vollständig remittiert, entsprechend einem Wert von 1 auf der Clinical Global Impression Scale (CGI-I). Postpartal konnte die erreichte Stabilität unter Risperidon erhalten werden.

### Anästhesie

Das regelhafte anästhesiologische Vorgehen für eine EKT außerhalb der Schwangerschaft beinhaltet eine intravenöse Narkoseinduktion. Das Narkotikum sollte einen raschen Wirkeintritt und eine kurze Wirkdauer besitzen und den induzierten Anfall möglichst gering beeinflussen. Zur Oxygenierung erfolgt nach Bewusstseinsverlust eine Beutel-Masken-Beatmung, seltener auch via Larynxmaske. Hierbei unterstützt eine moderate Hyperventilation eine angemessene Krampfaktivität. Eine endotracheale Intubation erfolgt in der Regel nicht, da der Eingriff an sich kein erhöhtes Aspirationsrisiko bedeutet. Vor Anfallsinduktion erfolgt eine Muskelrelaxation mittels eines kurz wirksamen Muskelrelaxans, in der Regel Succinylcholin [9].

Ab der 12. SSW besteht aufgrund einer verzögerten Magenentleerung ein erhöhtes Aspirationsrisiko. Zudem werden bereits in den ersten SSW die Schleimhäute vermehrt durchblutet mit Blutungs- und Ödemneigung der Schleimhäute im Bereich der oberen Atemwege und höherer Inzidenz für einen schwierigen Atemweg und erschwerter endotrachealer Intubation [22]. Weiterhin besteht bei Schwangeren eine niedrigere funktionelle Residualkapazität infolge des erhöhten intraabdominellen Drucks. Bei der Patientin wurde daher eine Nüchternheitszeit von sechs Stunden vor EKT streng eingehalten. Zur Pufferung des Magensafts erhielt sie zudem 30 ml 0,3-molares Natriumcitrat oral vor Narkoseeinleitung, zur Prophylaxe einer Hypersalivation Glycopyrroniumbromid i.v. (0,2 mg).

Die Einleitung der Allgemeinanästhesie erfolgte im Zentral-OP in Notsectiobereitschaft [10]. Es erfolgte eine leichte Linkslagerung und Optimierung des intravasalen Volumenstatus vor Beginn der EKT. Zielparameter war ein arterieller Mitteldruck von mindestens 65 mm Hg. In Rückenlage besteht ab der 16.–20. SSW das Risiko eines Vena-cava-inferior-Kompressionssyndroms durch den graviden Uterus. Eine Kompression der Aorta abdominalis wiederum kann eine verminderte uterine Durchblutung mit möglicher fetaler Asphyxie bedingen. Die erwähnte Lagerung und Optimierung reduziert diese Risiken. Bei Bedarf wurde der Blutdruck durch Gabe von Akrinor® (ratiopharm GmbH, Ulm, Deutschland) gesteigert.

Nach Präoxygenierung (exspiratorische Sauerstoffkonzentration >75 %) erfolgte die Narkoseinduktion durch die i.v.-Gabe von Methohexital (initial 1 mg/kg Körpergewicht, im Verlauf benötigte die Patientin eine Dosissteigerung auf 1,6 mg/kg). Mit Erreichen einer ausreichenden Narkosetiefe folgte die i.v.-Gabe von Succinylcholin (1,5 mg/kg Körpergewicht). Neben der Schwangerschaft bestanden bei der Patientin keine Hinweise auf einen schwierigen Atemweg. Zugunsten einer kürzeren Narkosedauer und einer geringeren Invasivität bei absehbar regelmäßiger EKT mit vulnerablen Schleimhäuten entschieden wir uns trotz des Aspirationsrisikos gegen eine Atemwegssicherung mittels Endotrachealtubus. Nach Narkoseinduktion wurde eine druckkontrollierte Maskenbeatmung mit einem maximalen Beatmungsdruck von 15 mbar durchgeführt. Anders als bei nichtschwangeren Patienten wurde auf eine Normoventilation geachtet. Eine exzessive Hyperventilation kann durch Entstehung einer respiratorischen Alkalose und dadurch verminderte Sauerstoffabgabe vom maternalen an das fetale Blut zu einer fetalen Hypoxie führen [18, 26].

Bei einmalig prolongiertem Anfallsgeschehen wurde Midazolam i.v. (1,5 mg) verabreicht. Dies beeinträchtige das Aufwachverhalten der Patientin nicht. Ein erweitertes Atemwegsmanagement war nicht nötig.

### Gynäkologie und Geburtshilfe

Einen wichtigen Aspekt in der Betreuung der Patientin stellte die regelmäßige Vorstellung in der Schwangerensprechstunde dar, durch die es gelang, ein Vertrauensverhältnis zwischen Patientin und behandelnden Gynäkologen/-innen aufzubauen. Dadurch konnten gemäß den aktuellen Mutterschaftsrichtlinien die vorgeschriebenen Vorsorgeuntersuchungen durchgeführt und im Mutterpass dokumentiert werden.

Aufgrund der Assoziation sowohl psychischer Erkrankungen als auch der EKT mit fetalen und maternalen Komplikationen (fetale Herzrhythmusstörungen, vorzeitiger Blasensprung, Uteruskontraktionen mit Eröffnung der Zervix, Plazentalösung und Frühgeburt [4, 26]) erfolgte ein intensives CTG-Monitoring je eine Stunde vor, kontinuierlich während sowie nach jeder EKT. Zudem wurden zum Ausschluss eines vorzeitigen Blasensprungs sowie einer Plazentalösung eine vaginale Inspektion mit Bestimmung des pH-Werts beziehungsweise ein Amni-Check und eine Zervixlängenmessung nach jeder EKT durchgeführt. Diese Maßnahmen sind essentiell zum Erkennen einer drohenden Frühgeburt.

Zur Verhinderung eines Atemnotsyndroms im Fall einer Frühgeburt erfolgte vor der ersten EKT mit 28 SSW eine Lungenreifeinduktion (2 × 12 mg Betamethason i.m. im Abstand von 24 h; [3]). Bedingt durch das Risiko einer Frühgeburt sollten die Planung und Durchführung jeder EKT bei schwangeren Patientinnen interdisziplinär und unter Notsectiobereitschaft erfolgen [10].

**Figure 1:**
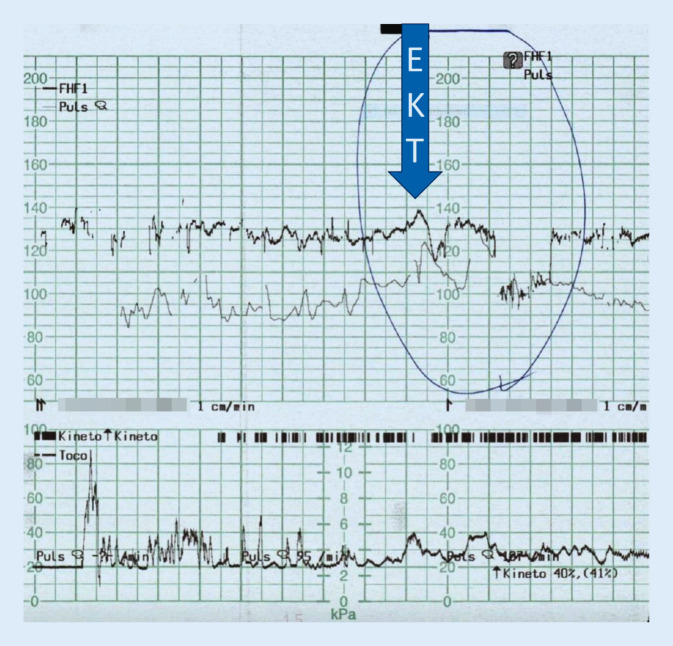

Hinsichtlich der einmalig aufgetretenen fetalen Bradykardie handelte es sich gemäß der CTG-Bewertung nach FIGO um eine variable Dezeleration <3 min, die definitionsgemäß als suspekt einzustufen ist. Da sich im Anschluss wieder eine normale fetale Herzfrequenz mit regelrechtem Oszillationsmuster darstellte, bestand kein Handlungsbedarf (Abb. 1).

Gemeinsam mit der Patientin entschieden wir uns aufgrund maternaler Erschöpfung mit ausgeprägter Angst vor einem Spontanpartus für eine primäre Sectio mit 37 SSW. Allgemein stellt die EKT keine Kontraindikation für eine Spontangeburt dar.

### Neonatologie

Anders als in klassischen Situationen der neonatologischen Beratungs- und Betreuungssituation konnte in diesem Fall kein adäquates pränatales „counseling“ der Mutter durchgeführt werden. Die durch die Grunderkrankung der Mutter eingeschränkte Kommunikationsmöglichkeit bei unzureichender Compliance bot keine Grundlage, über die etwaigen Risiken einer schwangerschaftsbegleitenden EKT für den Fetus bzw. das Neugeborene hinreichend aufzuklären. Daher erfolgte pränatal prioritär die Bereitstellung einer dem aktuellen Reifegrad des Fetus angepassten Erstversorgungsumgebung in den jeweiligen Operationseinheiten. Durch den Einsatz einer vollständig mobilen Erstversorgungseinheit als Inkubatormikroumgebung (Giraffe Omnibed-Shuttle® Einheit, Fa. GE) konnte auf die variable Gesundheitssituation der Mutter und den jeweiligen Therapieort der EKT kurzfristig eingegangen werden.

Bereits während der pränatalen Therapiephase ist zudem eine interdisziplinäre Planung der zu erwartenden Betreuungssituation des Neugeborenen durchzuführen. Ein Bindungsaufbau zwischen Mutter und Kind muss koordiniert und kann stufenweise und unter Wahrung der Sicherheit des Kindes auch im Setting einer Neugeborenen-IMC-Station etabliert werden.

## Ergebnisse

### Klinischer Fall

Im dargestellten Fall kam es unter EKT zu einer Remission des schizomanischen Syndroms inklusive des eigen- und fremdgefährdenden Verhaltens. Ein prolongierter Anfall der Patientin unter EKT konnte unkompliziert pharmakologisch beendet werden, eine einmalige kurzfristige fetale Bradykardie sistierte spontan, ansonsten traten keine relevanten unerwünschten Wirkungen auf. Das Kind wurde mittels geplanter Sectio mit 37 SSW gesund geboren und zeigte nach neonatologischer Untersuchung während des kurzfristigen stationären Aufenthalts keine organischen Auffälligkeiten und eine reife- und altersentsprechende Entwicklung.

### Vorschläge zum interdisziplinären Management bei EKT in der Schwangerschaft

Basierend auf den Erfahrungen der erfolgreichen Behandlung im oben dargestellten Fall sowie den zitierten systematischen Übersichtsarbeiten sollen nachfolgend tabellarisch Behandlungsvorschläge für die Durchführung der EKT in der Schwangerschaft formuliert werden (Tab. 2).

**Table 2:** 

Fachgebiet	Behandlungsvorschlag
Psychiatrie	Aufklärung über Diagnose, Behandlungsindikation, Therapieoptionen unter besonderer Berücksichtigung der Sicherheit für Mutter und Fetus. Abwägen der Chancen und Risiken der Therapie mit den nachteiligen Effekten der unbehandelten psychischen Störung
	Die Auswahl der Behandlungsparameter (Elektrodenposition, Stimulationsdosis, Pulsbreite etc.) unterscheidet sich bei Schwangeren nicht von den allgemeinen Prinzipien [8]
	Interdisziplinäre Durchführung mit Präsenz von Psychiatrie, Anästhesie, Gynäkologie/Geburtshilfe, Neonatologie
Gynäkologie/Geburtshilfe	*Vor Beginn der EKT-Serienbehandlung*
	Adäquate präpartale Versorgung: – Anlegen bzw. Vervollständigen des Mutterpasses – regelmäßige Vorstellung in Schwangerensprechstunde – frühzeitige Planung des Geburtsmodus zwischen Patientin, Geburtshilfe und Psychiatrie (Spontanpartus versus primäre Sectio; Spontanpartus bei guter Compliance möglich und primär angestrebt)
	Qualifizierter Ultraschall mit Bestimmung von – fetalem Gewicht – Fruchtwassermenge – plazentarer Versorgung (Dopplersonographie) – Zervixlänge (Verkürzung und damit verbundenes Frühgeburtsrisiko)
	CTG zur Ermittlung des fetalen Zustands (Herzfrequenz und Kindsbewegungen) sowie maternaler Kontraktionen
	Lungenreifeinduktion zwischen der 24+0. SSW und 34+0. SSW (2 × 12 mg Betamethason im Abstand von 24 h) zur Risikoreduktion eines „respiratory distress syndrome“
	*Während bzw. nach jeder EKT*
	Notsectiobereitschaft
	CTG-Monitoring 1 h vor, kontinuierlich während und 1 h nach EKT
	Vaginale Inspektion zum Ausschluss von Blutung bzw. Blasensprung (pH-Indikatorpapier bzw. Amni-Check)
Anästhesiologie	*Sorgfältige Anamnese*
	Hinweise auf eine erschwerte Atemwegssicherung (Mallampati-Klasse, Mundöffnung, Halswirbelsäulenbeweglichkeit, thyreomentale Distanz, bekannter schwieriger Atemweg in vorhergehenden Narkosen, Narkoseausweis vorhanden?)
	*Vor Beginn der EKT*
	Nüchternheitszeiten strikt einhalten
	Gabe von Natriumcitrat per os
	Frühzeitiges Anlegen eines i.v.-Zugangs und Gabe von kristalloider Infusion
	Gabe von Glycopyrroniumbromid i.v. vor Narkoseinduktion
	*Während EKT*
	Leichte Linkslagerung
	Suffiziente Präoxygenierung (exspiratorische Sauerstoffkonzentration >75 %)
	Normoventilation via Beutel-Masken-Beatmung (P_insp_ <20 mbar), Muskelrelaxation
	Engmaschige Blutdrucküberwachung, ggf. Gabe von Akrinor® (arterieller Mitteldruck >65 mm Hg)
	*Nach EKT*
	Nebenwirkungsmanagement: – Bei postinterventionellem Kopfschmerz Paracetamol als Mittel der Wahl. Alternativ Ibuprofen bis zur 28. Schwangerschaftswoche – Bei Übelkeit ist Meclozin Mittel der Wahl (über Auslandsapotheken erhältlich). Alternativ vorübergehend Dimenhydrinat (nicht im 3. Trimenon bei vorzeitiger Wehentätigkeit)
Neonatologie	Sicherstellung der Verfügbarkeit einer Maximalversorgung in jeder SSW inkl. adäquater Personalausstattung
	Nutzung einer mobilen Versorgungs- und Transporteinheit bei wechselnden Interventionsorten (integrierte Erstversorgungs- und Transporteinheit inkl. T‑Stück, Respirator und Gasversorgung)
	Kenntnis über die während der EKT genutzten Anästhetika im Falle einer Sectio caesarea (Apnoerisiko des Neugeborenen)
	Benennung eines klinischen Teams (pflegerisch & ärztlich), welches die Interventionen möglichst kontinuierlich koordiniert/begleitet

## Diskussion

Unser Fallbericht bestätigt, dass die EKT auch in der Schwangerschaft eine gut wirksame sowie für Mutter und Fetus sichere und verträgliche Therapieoption darstellen kann. Prinzipiell deckt sich diese Schlussfolgerung mit der verfügbaren Literatur. Sämtliche aktuellen deutschen Leitlinien für die Indikationen unipolare Depression, bipolare affektive Störung und Schizophrenie [5–7] benennen die EKT als therapeutische Option in der Schwangerschaft, jedoch mit der Einschränkung einer engen Indikationsstellung, sorgfältiger Nutzen-Risiko-Abwägung sowie Vorsichtsmaßnahmen („in Zentren der Maximalversorgung mit entsprechender Erfahrung in der Durchführung der EKT“ [7]). Diese Empfehlungen basieren auf vier systematischen Reviews [1, 15, 18, 21], die sich in ihrem methodischen Vorgehen relevant unterscheiden. Selbst zwei neuere und nahezu zeitgleich publizierte Arbeiten [15, 21] kommen aufgrund unterschiedlicher Einschlusskriterien und verschiedener Ansätze zur Bewertung von Komplikationen zu unterschiedlichen Risikoeinschätzungen [23]. Leiknes et al. [15] ließen in ihre Risikobewertung alle sog. „adverse events“ einfließen (d. h. alle unerwünschten Ereignisse unabhängig von einer möglichen Kausalität) und kommen zu einer sehr restriktiven Empfehlung der EKT bei Schwangerschaft im Sinne einer Ultima Ratio. Hingegen nahmen Pompili et al. [21] den Versuch einer Kausalitätsbeurteilung vor und fanden für viele Ereignisse keinen Zusammenhang mit der EKT. Entsprechend positiver fällt die Beurteilung der EKT aus als effektive therapeutische Option in der Schwangerschaft mit geringen Risiken.

Letztlich sind die absoluten und relativen Risiken der EKT in der Schwangerschaft nicht exakt bezifferbar, da kontrollierte Studien, die mittels identisch schwer erkrankter Vergleichspopulationen die Effekte der psychiatrischen Störung von denen der Behandlung differenzieren könnten, fehlen. Eine ohne Kausalitätsprüfung vorgenommene Angabe der Häufigkeit unerwünschter Ereignisse bei EKT in der Schwangerschaft wird diese jedoch überschätzen, da die EKT-assoziierten „adverse events“ [23] qualitativ weitgehend deckungsgleich mit den Risiken einer schwerwiegenden psychischen Erkrankung in der Schwangerschaft [14, 28] sind (Tab. 1). Einigkeit sollte bestehen, dass durch die elektrische Stimulation keine teratogenen Effekte zu erwarten sind. Eine theoretische Arbeit zeigte zudem, dass die modellhaft berechneten Feldstärken im Bereich des fetalen Gehirns selbst bei maximalen Geräteeinstellungen unter den Grenzwerten für elektromagnetische Felder liegen [13].

Aus neonatologischer Sicht stellt am ehesten die Anästhesie ein potenzielles Risiko für den Fetus dar. Zur Anwendung im letzten Trimenon der Schwangerschaft liegt ein Warnhinweis der FDA bezüglich der Hirnentwicklung des Fetus und des Früh- und Neugeborenen vor [11], der jedoch zugleich äußert, dass einzelne bzw. kurze (<3 h) Anästhesien wahrscheinlich keinen negativen Effekt auf Verhalten und Lernen des Kindes haben. Die FDA ergänzte, dass schwangere Frauen bei gegebener Indikation und kurzer Anästhesiedauer (<3 h) einen notwendigen Eingriff nicht verzögern sollten [12]. Letztlich ist die klinische Evidenz unzureichend, um einen neurotoxischen Effekt prolongierter oder wiederholter Anwendung von Anästhetika in Schwangerschaft und Neugeborenenperiode beim Menschen ausschließen zu können [25]. Eine größere prospektive Multicenter-RCT ließ keine signifikant häufigeren Auffälligkeiten mit 2 bzw. 5 Jahren nach Narkoseinterventionen erkennen [17], wobei diese Studie keine Behandlung von Schwangeren und keine Mehrfachnarkosen beinhaltete und somit nur eingeschränkt übertragbar ist.

Aufgrund der heterogenen Datenbasis und Schlussfolgerungen der Übersichtsarbeiten zu EKT in der Schwangerschaft ist es weiterhin sinnvoll, entsprechende Fallberichte und Fallserien zu publizieren. Zur besseren kausalen Einordnung möglicher unerwünschter Ereignisse bei Mutter und Fetus bzw. Kind wären Fall-Kontroll-Studien wünschenswert, die aus methodischen Gründen jedoch schwer zu realisieren sind.

In Übereinstimmung mit den Leitlinien besteht unter den Autoren interdisziplinärer Konsens hinsichtlich einer engen Indikationsstellung zur EKT in der Schwangerschaft mit besonders sorgfältiger Betrachtung von Diagnose/Indikation, individueller Nutzen-Risiko-Abwägung und therapeutischen Alternativen. Ist dies gegeben und werden die o. g. interdisziplinären Behandlungsvorschläge beachtet, gibt es jedoch nach unserer übereinstimmenden Beurteilung keinen Grund, bei vorliegender Schwangerschaft auf die EKT zu verzichten. Kommt wie im vorliegenden Fall auch noch eine unmittelbare Gefährdung für Mutter und Fetus durch Erregungszustände und selbstverletzendes Verhalten hinzu, sollte das Nutzen-Risiko-Verhältnis deutlich zugunsten der EKT ausfallen.

## Fazit für die Praxis

- Die EKT kann auch in der Schwangerschaft eine wirksame und sichere Therapieoption bei schweren psychischen Störungen sein.
- Eine Indikation zur EKT in der Schwangerschaft besteht bei schwerer affektiver oder psychotischer Symptomatik, daraus resultierender Gefährdung von Mutter und/oder Fetus sowie fehlender Wirksamkeit, Verträglichkeit oder Umsetzbarkeit anderer Therapien.
- Die kasuistisch beschriebenen unerwünschten Ereignisse entsprechen qualitativ den allgemeinen Risiken bei schwerer psychischer Störung in der Schwangerschaft und sind daher in ihrer Kausalität unklar.
- Ein abgestimmtes interdisziplinäres Management zwischen Psychiatrie, Gynäkologie, Anästhesiologie und Neonatologie ist grundlegend für die sichere Durchführung.
- Die Publikation von weiteren Fallberichten und -serien erscheint zur Verbesserung der Evidenzgrundlage sinnvoll.
